# Environmental management of asthma in clinical practice: Results from the 2012 National Ambulatory Medical Care Survey

**DOI:** 10.1016/j.jacig.2023.100192

**Published:** 2023-11-22

**Authors:** Paivi M. Salo, Lara J. Akinbami, Michelle M. Cloutier, Jesse C. Wilkerson, Kurtis S. Elward, Jacek M. Mazurek, Gregory B. Diette, Tracey A. Mitchell, Sonja Williams, Darryl C. Zeldin

**Affiliations:** a Division of Intramural Research, National Institute of Environmental Health Sciences, National Institutes of Health, Research Triangle Park, NC; b National Center for Health Statistics, Centers for Disease Control and Prevention, Hyattsville, Md; c United States Public Health Service, Rockville, Md; d Department of Pediatrics, University of Connecticut, Farmington, Conn; e Social & Scientific Systems, a DLH Holdings company, Durham, NC; f Department of Family Medicine and Population Health, The Virginia Commonwealth University, Richmond, Va; g National Institute for Occupational Safety and Health, Centers for Disease Control and Prevention, Morgantown, WV; h Department of Medicine, School of Medicine, Johns Hopkins University, Baltimore, Md; i Environmental Protection Agency, Washington, DC

**Keywords:** Asthma, asthma guidelines, environmental control, guideline implementation, national survey

## Abstract

**Background:**

The National Asthma Education and Prevention Program guidelines emphasize environmental control as an integral part of asthma management; however, limited national-level data exist on how clinicians implement environmental control recommendations.

**Objective:**

We analyzed data on clinicians’ self-reported use of recommended environmental control practices in a nationally representative sample (n = 1645) of primary care physicians, asthma specialists, and advanced practice providers from the National Asthma Survey of Physicians, a supplemental questionnaire to the 2012 National Ambulatory Medical Care Survey.

**Methods:**

We examined clinician and practice characteristics as well as clinicians’ decisions and strategies regarding environmental trigger assessment and environmental control across provider groups. Regression modeling was used to identify clinician and practice characteristics associated with implementation of guideline recommendations.

**Results:**

A higher percentage of specialists assessed asthma triggers at home, school, and/or work than primary care or advanced practice providers (almost always: 53.6% vs 29.4% and 23.7%, respectively, *P* < .001). Almost all clinicians (>93%) recommended avoidance of secondhand tobacco smoke, whereas recommendations regarding cooking appliances (eg, proper ventilation) were infrequent. Although assessment and recommendation practices differed between clinician groups, modeling results showed that clinicians who reported almost always assessing asthma control were 5- to 6-fold more likely to assess environmental asthma triggers. Use of asthma action plans was also strongly associated with implementation of environmental control recommendations.

**Conclusions:**

Environmental assessment and recommendations to patients varied among asthma care providers. High adherence to other key guideline components, such as assessing asthma control, was associated with environmental assessment and recommendation practices on environmental control.

Control of environmental factors is an important part of asthma management and can reduce asthma morbidity and mortality.^1^, ^2^, ^3^, ^4^ Asthma guidelines recommend avoidance and reduction of exposures that exacerbate the disease, including exposures to allergens, tobacco smoke, air pollution, and other irritants.^5^^,^^6^ Although environmental management of asthma is endorsed in the guidelines, limited national-level data on how clinicians implement these guideline recommendations are available.

The National Asthma Survey of Physicians (NAS), a one-time supplemental questionnaire to the 2012 National Ambulatory Medical Care Survey (NAMCS), was the first survey to collect nationally representative data on clinicians’ opinions, self-efficacy (ie, clinician’s self-reported level of confidence in implementing specific guideline recommendations), and clinical decisions regarding asthma care and key components of the 2007 National Asthma Education and Prevention Program’s (NAEPP) Expert Panel Report 3 (EPR-3). The NAS data offered a unique opportunity to investigate whether clinicians’ practices align with the evidence-based recommendations highlighted in the guidelines.^7^, ^8^, ^9^ Understanding clinicians’ attitudes, skills and guideline usage may improve guideline implementation as well as patient care. Here, we focus on environmental assessment and recommendations on environmental control outlined in the EPR-3 Guideline Component 3: Assessment and Control of Environmental Factors. We compared clinicians’ decisions and strategies regarding environmental control measures among primary care physicians, asthma specialists, and advanced practice providers (physician assistants, nurse practitioners, and nurse midwives), given that the guidelines were intended to stimulate adoption of the recommendations among all clinicians who see asthma patients.^6^ We also used regression models to determine clinician and practice characteristics that were associated with self-reported guideline implementation. We characterized similarities and differences in environmental management of asthma across clinician groups to help inform future efforts to improve adoption and implementation of guideline recommendations.

## Methods

### Study population and data collection

Data for this study were collected through the 2012 NAMCS, a health care survey administered by the National Center for Health Statistics (NCHS) to evaluate the utilization and provision of ambulatory care services in the United States, and released in 2017.^10^^,^^11^ The NAMCS sampling frame consisted of a nationally representative sample of clinicians in office- and community health center (CHC)-based patient care, excluding specialty physicians in anesthesiology, radiology, and pathology, and those aged 85 years and over. Advanced practice providers were included and were sampled only within CHCs. A supplemental clinician questionnaire (NAS) was included in the 2012 NAMCS survey cycle to assess the use and acceptance of the EPR-3 recommendations.^12^ Allergists and pulmonologists were oversampled to ensure a sufficient sample size for asthma specialists. All clinicians who responded affirmatively to the question “Do you see any patients for whom you provide asthma diagnosis, education and/or ongoing clinical management?” received the NAS questionnaire and were included in this study.^13^ The NCHS ethics review board approved the NAMCS protocol, and informed consent was obtained from all participating clinicians. Additional information on the survey design and implementation of NAMCS and NAS is published elsewhere.^10^^,^^14^

The unweighted and weighted response rates for the overall NAS sample were 38% and 28%, respectively, comparable with other physician surveys.^15^, ^16^, ^17^ Of the 1726 respondents, we excluded clinicians who practiced in specialties unlikely to regularly manage asthma (n = 49), nonclinical respondents (n = 15), and those with missing demographic data (n = 17). The final sample of 1645 clinicians included 1069 primary care physicians (office- and CHC-based general/family practitioners, internists, pediatricians, and obstetricians), 233 asthma specialists (office-based allergists and pulmonologists), and 343 advanced practice practitioners (referred to as midlevel providers in the 2012 NAMCS questionnaire) from CHCs.

### Outcome and independent variables of questionnaire data

Participants reported clinician and practice characteristics, including clinician specialty, age and sex, census region and level of urbanization of practice location, practice ownership, age group served by the practice, weekly asthma patient volume, type of patient record/management system, and use of asthma-specific encounter forms. Primary care and CHC clinicians also provided information on specialist referral frequency. Questionnaire data on asthma care were categorized according to the EPR-3 guidelines’ core components: (1) assessment and monitoring of asthma severity and control, (2) patient education, (3) assessment and control of environmental factors, and (4) pharmacologic treatment of asthma (see Table E1 in the Online Repository at www.jaci-global.org).

Level of implementation of guideline recommendations was determined on the basis of the percentage of asthma visits in which each recommendation was followed using a Likert-type scale, as follows: almost always (75-100%), often (25-75%), sometimes (1-24%), and never (0%). As a result of low response frequencies, the categories “sometimes” and “never” were combined. We also created 3 dichotomous index variables. The first one, a variable combining responses to how frequently clinicians assessed impairment and risk, reflected implementation of asthma control recommendations (Table E1). This asthma control index identified clinicians who reported almost always assessing impairment and risk, either with specific questions or a control assessment tool (ie, clinicians who either asked about patient’s ability to engage in daily activities, frequency of daytime and nighttime symptoms, and frequency of rescue inhaler use, or used a control assessment tool to assess impairment, and who also assessed risk by querying about the frequency of emergency department/urgent care visits for asthma and the frequency of exacerbations requiring oral steroids). The 2 additional index variables reflected strong overall agreement and self-efficacy with EPR-3 guideline recommendations (see Table E2 in the Online Repository at www.jaci-global.org). The agreement index dichotomized responses to all 5 questions about agreement as “strongly agree” versus all other responses (agree, neutral, disagree, strongly disagree), while the self-efficacy index dichotomized responses to all 5 questions about self-efficacy as “very confident” versus all other responses.

Outcomes focused on clinician implementation of the EPR-3 Guideline Component 3: Assessment and Control of Environmental Factors (Table E1). Clinicians were asked how often they assessed patient’s triggers at home, school, and work, and whether they performed allergy testing. We created a composite outcome combining the assessment of asthma triggers at home, school, and work to reflect a thorough assessment of triggers during asthma visits. Additional questions asked about recommendations to patients regarding indoor and outdoor triggers. These questions focused on recommendations geared toward specific patient populations (eg, patients with dust mite allergy) and asked respondents whether recommendations were given to most patients, were given only to those with risk factor or factors, or were rarely or never given.

### Statistical analysis

Descriptive statistics were used to summarize clinician and practice characteristics and outcome measures. Differences between the 3 clinician groups across the response categories were assessed by chi-square test, with *P* < .05 considered statistically significant. Only significant comparisons are mentioned here. Estimates with lower precision (ie, standard error > 30% of estimate) are noted.

Multivariable regression modeling was used to identify clinician and practice characteristics associated with self-reported implementation of guideline recommendations on environmental control. We used binomial logistic regression to identify characteristics associated with frequent trigger assessment (ie, assessment of environmental triggers at home, school, and work conducted almost always during/75-100% of asthma visits). Clinician-reported recommendations regarding environmental control practices were modeled separately using generalized multinomial logistic regression models. We chose a data-driven approach and evaluated independent variables in bivariate analyses before modeling. Those with bivariate association *P* < .25 were selected for the initial full model, and then backward elimination variable selection was iteratively used until all remaining independent variables in each final model had type III *P* < .05. Only adjusted associations are discussed here in the text. Missing observations were excluded if the missing rate was low (<5%); otherwise, a separate “missing” category was included to maximize observations in the analysis. To avoid unstable model estimates, variables with low cell counts (<5 observations per category) were either excluded or categories were collapsed when possible.

Descriptive analyses and modeling were performed by SAS v9.4 (SAS Institute) and SUDAAN 11.0. (RTI International). To account for the complex survey design, unequal probability of selection, and nonresponse, sampling weights and design variables were applied to all analyses.

## Results

### Demographic and asthma care characteristics and asthma control, agreement, and self-efficacy indices

Demographic and asthma care characteristics (Tables I and II) among clinicians who cared for asthma patients varied significantly by clinician group. Higher percentages of CHC advanced practice providers were generally younger, female, and saw patients of all ages than asthma specialists and primary care clinicians (Table I). In contrast, specialists and primary care clinicians were more likely to work in private settings and in large metropolitan areas. Specialists tended to have higher weekly asthma patient volumes and more frequently used patient information management systems (electronic or nonelectronic) than primary care and advanced practice providers.

**Table I:** Demographic characteristics of study population by clinician group (weighted percentages), 2012 NAS

Characteristic	All clinicians (N = 1645)		Primary care (n = 1069)	Asthma specialist (n = 233)	CHC advanced practice (n = 343)	*P* value∗
	No.	% (SE)	% (SE)	% (SE)	% (SE)	
Clinician age						<.001
60+ years	366	24.1 (1.9)	24.4 (2.2)	36.5 (4.0)	12.8 (2.6)	
40-59 years	907	60.3 (2.1)	62.3 (2.4)	54.0 (4.1)	43.3 (4.3)	
<40 years	372	15.6 (1.4)	13.3 (1.5)	9.5 (2.3)	43.9 (4.9)	
Clinician sex						<.001
Female	772	40.0 (2.0)	37.3 (2.3)	15.7 (2.7)	84.1 (2.7)	
Male	873	60.0 (2.0)	62.7 (2.3)	84.3 (2.7)	15.9 (2.7)	
Census region						.006
Northeast	255	20.7 (0.9)	21.1 (1.0)	21.1 (2.0)	16.2 (2.7)	
Midwest	400	19.5 (0.6)	19.5 (0.7)	17.8 (1.5)	20.0 (2.1)	
South	553	30.2 (0.8)	30.2 (0.9)	38.0 (2.7)	25.2 (2.6)	
West	437	29.7 (0.8)	29.2 (1.0)	23.2 (1.8)	38.7 (3.6)	
Level of urbanization						<.001
Nonmetro	376	14.3 (1.1)	13.6 (1.2)	6.3 (2.2)†	27.3 (3.7)	
Medium/small metro	532	28.8 (1.9)	28.4 (2.0)	27.2 (3.7)	34.5 (5.1)	
Large metro	737	56.8 (1.9)	58.0 (2.1)	66.5 (3.8)	38.1 (5.0)	
Ownership of practice						<.001
Private	653	61.7 (1.8)	66.1 (2.1)	84.4 (2.8)	0	
CHC	688	16.0 (0.8)	10.3 (0.8)	0	86.1 (2.8)	
HMO, academic center, other	194	16.1 (1.6)	17.3 (1.8)	12.0 (2.5)	6.9 (2.2)	
Missing	110	6.2 (1.1)	6.3 (1.3)	3.6 (1.4)†	7.0 (1.8)	
Patient population						<.001
Age specific (pediatric/adult only)	649	47.5 (2.2)	48.7 (2.5)	58.8 (3.6)	27.1 (4.7)	
Patients of all ages	996	52.5 (2.2)	51.3 (2.5)	41.3 (3.6)	72.9 (4.7)	
Weekly asthma patient volume						<.001
<3 patients	295	20.1 (1.9)	21.1 (2.1)	8.3 (2.3)	17.0 (3.0)	
3-12 patients	826	47.0 (2.1)	47.7 (2.3)	30.2 (3.8)	50.4 (4.2)	
13+ patients	388	21.7 (1.8)	19.7 (2.0)	50.9 (4.0)	22.2 (4.4)	
Missing	136	11.3 (1.6)	11.4 (1.8)	10.7 (2.7)	10.4 (3.5)†	
Patient information management						.004
No system	210	17.9 (1.9)	19.1 (2.1)	7.5 (2.2)	12.5 (2.7)	
Non-EMS	222	15.8 (1.6)	15.8 (1.8)	22.7 (3.7)	11.3 (3.5)†	
EMS	1,108	59.4 (2.2)	58.8 (2.5)	61.1 (4.1)	64.5 (5.1)	
Missing	105	6.9 (1.2)	6.3 (1.3)	8.8 (2.0)	11.7 (3.6)†	

Source: National Center for Health Statistics, 2012 NAS: National Ambulatory Medical Care Survey. *EMS,* Electronic management system; *HMO,* health maintenance organization; *SE,* standard error.

∗ Chi-square test for difference between clinician groups.

† SE > 30% of estimate (unreliable estimate).

**Table II:** Asthma care characteristics by clinician group (weighted percentages), 2012 NAS

Characteristic	All clinicians (N = 1645)		Primary care (n = 1069)	Asthma specialist (n = 233)	CHC advanced practice (n = 343)	*P* value∗
	No.	% (SE)	% (SE)	% (SE)	% (SE)	
Asthma encounter form						.138
No form	377	26.1 (2.0)	26.8 (2.2)	22.8 (3.7)	21.2 (4.4)	
Form not used	323	20.9 (1.9)	21.3 (2.1)	23.0 (3.6)	15.7 (3.0)	
Form used	919	52.0 (2.2)	51.1 (2.5)	53.9 (4.1)	59.8 (4.3)	
Missing	26	1.0 (0.4)†	0.8 (0.4)†	0.3 (0.3)†	3.2 (1.1)†	
Asthma control index‡						<.001
Yes	694	39.5 (2.2)	37.7 (2.4)	75.2 (4.0)	33.9 (4.5)	
No	931	58.8 (2.2)	60.4 (2.5)	25.7 (4.0)	65.7 (4.5)	
Missing	20	1.7 (0.5)	2.0 (0.6)†	0	0.4 (0.3)†	
Documentation of asthma control						<.001
Almost always (75-100%)	605	32.2 (1.9)	29.5 (2.2)	74.4 (4.0)	32.3 (5.0)	
Often (25-74%)	620	40.2 (2.2)	42.2 (2.5)	19.9 (3.6)	32.8 (4.4)	
Sometimes/never (0-24%)	325	20.6 (1.8)	21.2 (2.1)	2.6 (1.2)†	26.6 (3.9)	
Missing	95	7.0 (1.3)	7.1 (1.4)	3.1 (1.7)†	8.3 (2.6)†	
Use of AAP						<.001
Almost always (75-100%)	320	16.9 (1.5)	15.6 (1.7)	30.6 (3.6)	21.2 (4.2)	
Often (25-74%)	486	30.3 (2.0)	30.3 (2.3)	32.7 (4.1)	28.9 (4.2)	
Sometimes/never (0-24%)	819	51.2 (2.2)	52.4 (2.5)	36.7 (4.4)	49.3 (4.9)	
Missing	20	1.6 (0.5)†	1.8 (0.5)	0	0.7 (0.4)†	
Agreement index§						.028
Strongly agree	257	12.9 (1.3)	11.5 (1.5)	27.9 (3.9)	18.0 (3.7)	
Not agree strongly	1385	86.7 (1.3)	88.1 (1.5)	72.1 (3.9)	82.0 (3.7)	
Missing	3	0.4 (0.3)†	0.5 (0.3)†	0	0	
Self-efficacy index§						<.001
Very confident	447	24.2 (2.0)	22.5 (2.3)	72.3 (3.9)	9.0 (2.3)	
Not very confident	1195	75.3 (2.0)	76.9 (2.3)	27.7 (3.9)	91.0 (2.3)	
Missing	3	0.5 (0.3)†	0.6 (0.4)†	0	0	
Referral to a specialist						.729
Almost always (75-100%)	97	8.1 (1.4)	8.3 (1.5)	NA	6.1 (2.1)†	
Often (25-74%)	392	28.2 (2.0)	28.0 (2.2)		30.4 (4.5)	
Sometimes/never (0-24%)	885	59.6 (2.2)	59.5 (2.4)		60.2 (4.7)	
Missing	38	4.1 (1.0)	4.2 (1.1)		3.2 (1.5)†	

Source: National Center for Health Statistics, 2012 NAS: National Ambulatory Medical Care Survey. *NA,* Not applicable (specialist referrals apply to primary care and CHC clinicians, and no specialist worked at a CHC); *SE,* standard error.

∗ Chi-square test for difference between clinician groups.

† SE > 30% of estimate (unreliable estimate).

‡ Clinician’s assessment of asthma control was ascertained with “almost always” responses to impairment- and risk-related questions: (clinician queried patient’s ability to engage daily activities, frequency of daytime and nighttime symptoms, and frequency of rescue inhaler use) *or* (clinician used control assessment tool) *and* (clinician asked about frequency of emergency department/urgent care visits for asthma and frequency of exacerbations requiring oral steroids).

§ Index variables reflect strong agreement with and/or high confidence in performing all 5 EPR-3 recommendations shown in Table E2.

Specialists tended to almost always assess (75.2%) and document (74.4%) asthma control compared to primary care and advanced practice providers (29.5-37.7%; Table II). Low percentages of providers almost always used asthma action plans (AAP): 15.6% of primary care providers, 21.2% of advanced practice providers, and 30.6% of specialists. Slightly over half (52.0%) of clinicians reported using asthma encounter forms. Referral patterns were similar among primary care and advanced practice providers; over one third (36.3%) of clinicians reported almost always or often referring patients to specialists. While agreement with selected key EPR-3 recommendations (usefulness of spirometry for asthma diagnosis, inhaled corticosteroid effectiveness, AAPs effectiveness, need for ≤6-month follow-up visits, and assessing severity for initial treatment) was generally higher among specialists, agreement with all 5 recommendations was not common, ranging from 11.5% to 27.9% across the groups. Most specialists (72.3%) reported high self-efficacy in managing asthma, whereas a minority of primary care and advanced practice providers (22.5% and 9.0%, respectively) expressed high confidence in implementing all 5 guideline recommendations (ie, using spirometry, assessing severity, prescribing inhaled corticosteroids, and stepping up and stepping down treatment).

### Assessment of asthma triggers and recommended environmental control practices

**Fig 1:**
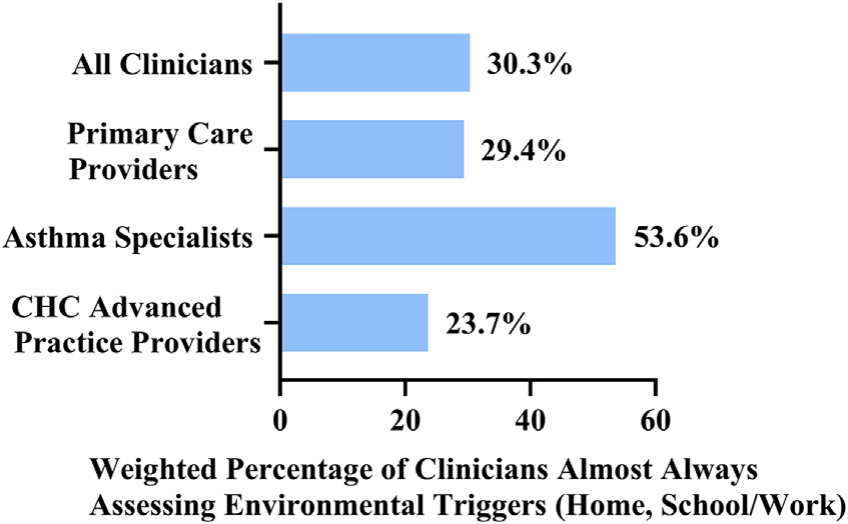
Weighted percentage of clinicians almost always assessing environmental triggers at home and school/work during asthma visits. Chi-square test for difference between clinician groups (primary care providers, asthma specialists, CHC advanced practice providers); *P* < .001.

**Fig 2:**
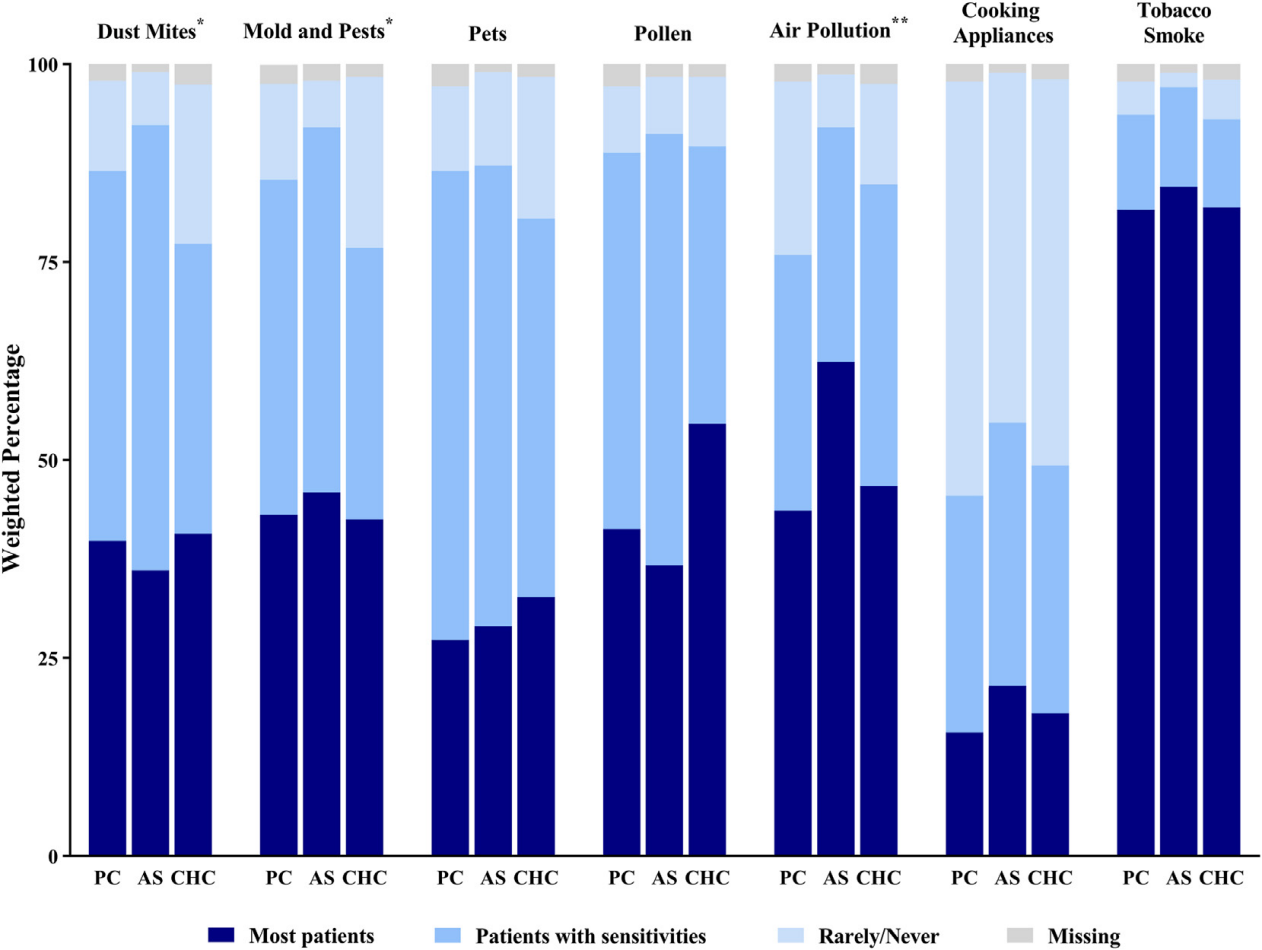
Weighted percentage of clinicians recommending environmental control practices by clinician group. Chi-square test for difference between groups (*AS,* asthma specialist; *CHC,* CHC advanced practice provider; *PC,* primary care provider); ∗*P* < .05, ∗∗*P* < .001.

Frequency of assessing environmental asthma triggers during patient visits varied considerably across clinician groups (Fig 1; see Table E3 in the Online Repository at www.jaci-global.org). The percentage of clinicians who almost always assessed triggers at home and at school or work varied from 23.7% of advanced practice providers to 53.6% of specialists. Similarly, nearly two thirds of asthma specialists (63.3%) reported almost always or often reviewing triggers in the AAP compared to half or less of primary care and advanced practice providers (45.9-50.1%) (Table E3). Objective assessment of patients’ sensitization status was largely performed by specialists; 67.3% of specialists conducted the assessment almost always or often versus 24.8% of primary care and 14.6% of CHC clinicians. Differences were also found on how clinicians advised their patients (Fig 2; see Table E4 in the Online Repository). While almost all clinicians (>93%) recommended avoidance of secondhand tobacco smoke, advice regarding cooking appliances (eg, exhaust ventilation) was given less frequently (<47%). Recommendation patterns for dust mite, mold, pest, and air pollution avoidance showed variation across the clinician groups. The percentage who recommended avoidance of air pollution to their patients ranged from a high of 92.0% among specialists to 75.9% among primary care physicians. Specialists and primary care providers recommended control measures for indoor allergens more frequently than advanced practice providers. However, there was no significant difference in frequency of recommended pollen avoidance. Pet removal recommendations also did not differ significantly across the groups; nearly 60% of specialists and primary care clinicians recommended removing pets from homes of patients with pet sensitivities, whereas 47.8% of advanced practice providers did so.

### Clinician and practice characteristics associated with implementation of guideline recommendations

**Fig 3:**
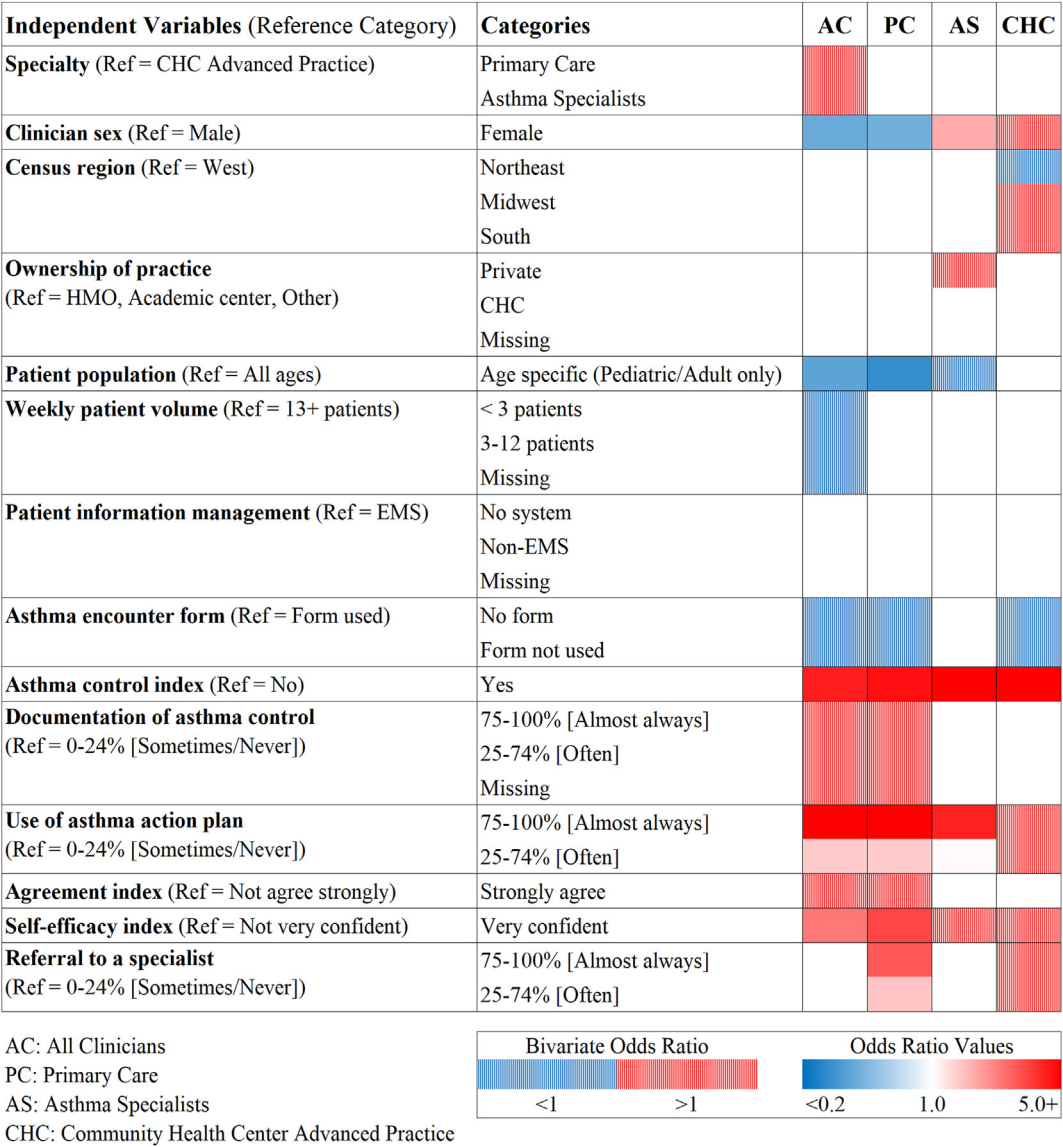
Factors associated with guideline implementation for environmental trigger assessment at home and school/work (assessment conducted almost always during asthma visits). *Solid red* or *blue* indicates direction of associations (*red,* positive; *blue,* negative; *P* < .05) when each of clinician/practice characteristic categories was compared to reference category adjusting for other independent variables in final group-specific models. *Dashed areas* indicate clinician/practice characteristics included in initial full models based on bivariate analysis results.

Fig 3 shows adjusted odds ratios (OR) for initial and final models for environmental trigger assessment and identifies factors that were associated with high assessment frequency (triggers assessed “almost always,” ie, 75-100% of asthma visits); numeric values are presented in Table E5 available in the Online Repository at www.jaci-global.org. Across all clinician groups, self-reported implementation of guideline recommendations on asthma control was consistently associated with 5- to 6-fold higher odds of environmental trigger assessment (ORs ranging from 4.78 to 6.12). Almost always using AAPs was strongly associated with higher odds of trigger assessment among asthma specialists (OR = 4.50; 95% confidence interval [CI], 1.73-11.73) and primary care clinicians (OR = 6.69; 95% confidence interval, 3.38-13.24). Female asthma specialists had higher odds of assessing environmental asthma triggers than male asthma specialists (OR = 2.32; 95% CI, 1.03-5.24), whereas the opposite was observed among primary care physicians (OR = 0.56; 95% CI, 0.33-0.95). High self-efficacy (OR = 3.93; 95% CI, 2.20-7.00) and referral frequency (OR = 3.62; 95% CI, 1.19-11.04) were associated with high assessment frequency among primary care clinicians, while the odds of assessment were lower for those who saw age-specific populations compared to those who provided care to patients of all ages (OR = 0.37; 95% CI, 0.22-0.63). The findings for the total population of clinicians largely reflected results of primary care providers who were a majority of the study population.

**Fig 4:**
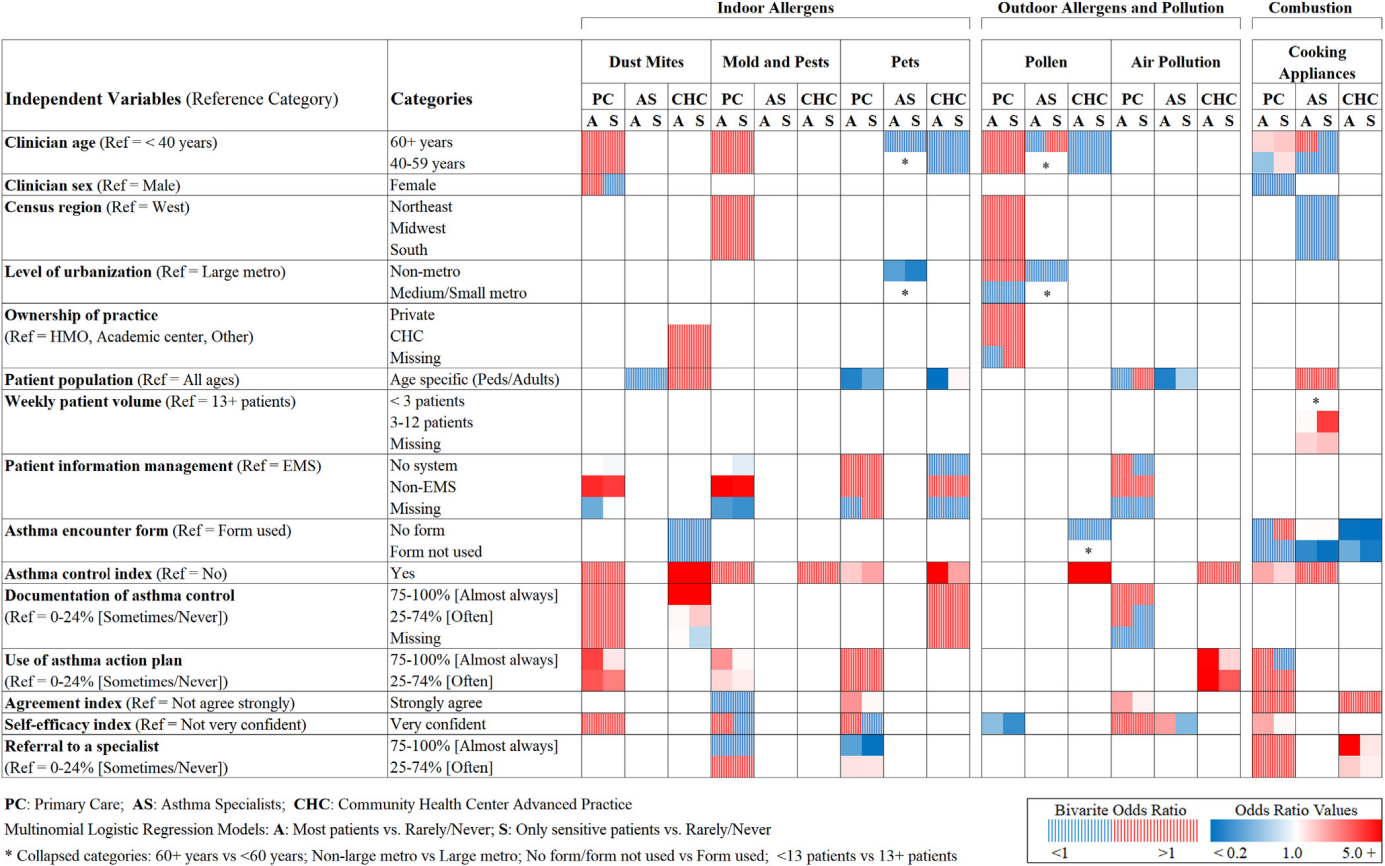
Factors associated with guideline implementation for environmental control recommendations by clinician group. *Solid red* or *blue* indicates direction of associations (*red,* positive; *blue,* negative; *P* < .05) when each of clinician/practice characteristic categories was compared to reference category adjusting for other independent variables in final group-specific models. *Dashed areas* indicate clinician/practice characteristics included in initial full models based on bivariate analysis results.

Fig 4 shows adjusted ORs for initial and final models for recommendation practices for each clinician group, comparing the odds of advising patients to implement environmental control measures (most patients and patients with sensitivities, separately) versus rarely or never recommending avoidance measures. Numeric results for Fig 4 and results for the total clinician population are included in the Online Repository available at www.jaci-global.org, see Fig E1 and Tables E6-E11. Recommendations related to secondhand tobacco smoke avoidance were excluded from modeling because almost all clinicians (>93%) adhered to the guideline recommendations. Characteristics associated with recommendation practices varied greatly by clinician group and recommendation type. Among primary care and CHC advanced practice providers, implementation of guideline recommendations on asthma control was associated with higher odds of providing advice on environmental control measures (Fig 4, Tables E6-E11). The odds of recommending control measures were higher when asthma control was almost always assessed during visits, ranging from 1.67 (95% CI, 1.02-2.75) among primary care clinicians for cooking appliances to 9.56 (95% CI, 1.73-52.99) among CHC advanced practice providers for pollen avoidance. Frequent AAP use was associated with increased odds of recommending dust mite, mold, pest, and air pollution avoidance, but strength of association varied by clinician group. The strongest associations were observed among CHC advanced practice providers for air pollution (OR = 5.36; 95% CI, 1.76-16.26) and among primary care clinicians for dust mite avoidance recommendations (OR = 3.99; 95% CI, 1.50-10.57). Pet removal was recommended less frequently by clinicians who provided care for age-specific populations than those who saw patients of all ages. Other clinician and practice characteristics, including clinician age, guideline agreement, self-efficacy, specialist referral, characteristics of the patient information management system, and the lack of use of asthma encounter forms, were less frequently associated with patient recommendations, and the observed patterns were less consistent. Because most asthma specialists recommended environmental control measures to their patients, few group-specific factors were associated with recommendation practices. A lower percentage of specialists in non–large metro areas, however, recommended pet removal, especially to pet-sensitive patients (OR = 0.31; 95% CI, 0.12-0.79), than specialists in large metro areas. Patient population characteristics, self-efficacy, and not using asthma encounter forms were also associated with specialists’ recommendations, but no consistent patterns were found. In the total clinician population (Fig E1, Tables E6-E11), clinician specialty and reported use of AAPs were most consistently associated with environmental control-related recommendations.

## Discussion

Control of environmental factors is one of the cornerstone components of the national asthma guidelines that underscore the importance of identifying and reducing exposures that increase asthma symptoms and precipitate asthma exacerbations.^6^^,^^18^ The NAS, the first survey that can be used to evaluate guideline implementation among clinicians at the national level, showed that environmental assessment and recommendation practices on environmental control varied considerably by clinician group. A higher percentage of asthma specialists assessed asthma triggers than primary care or advanced practice providers. Yet a large percentage of clinicians did not assess asthma triggers frequently; 46% to 76% of clinicians, depending on clinician type, reported not assessing triggers almost always during asthma visits. Clinician groups advised patients differently regarding several triggers, but similarities were also found. Almost all clinicians recommended avoiding secondhand tobacco smoke, and most clinicians reported recommending pet removal, especially from homes of patients with pet sensitivities. In contrast, advice regarding cooking appliances was infrequent. Modeling results suggested that high frequency of implementation of other key components of the guidelines, including the use of AAPs and asthma control recommendations, was associated with more consistent implementation of the EPR-3 recommendations regarding environmental control.

A major finding was the nearly opposite patterns for 2 important sources of indoor air irritants: almost all clinicians recommended avoidance of secondhand tobacco smoke, but less than half advised their patients on cooking appliances. The guidelines encourage clinicians to advise patients to avoid, if possible, exposure to gas stoves and appliances that are not vented outside, as well as fumes from wood-burning appliances and fireplaces, sprays, or strong odors.^6^ Cooking and cooking appliances can be substantial sources of indoor air pollutants, especially in smaller kitchens and in homes with poor ventilation or without vented range hoods.^19^, ^20^, ^21^ Using gas stoves without use of a range hood or with poor ventilation, for example, can increase indoor levels of NO_2_ above thresholds associated with respiratory symptoms and asthma exacerbations (ie, indoor levels exceed US ambient air standards).^20^^,^^22^

Most clinicians recommended pet removal to patients with pet sensitivities, but a relatively high proportion of clinicians, up to one third, reported recommending pet removal for all patients. Given that allergy testing was largely performed by specialists, this is not necessarily surprising, although the guidelines recommend allergen mitigation interventions to patients who are both exposed to and either sensitized to or develop symptoms on exposure to specific allergens.^6^^,^^18^ Yet specialists outside large metro areas less frequently recommended pet removal than those in urban areas, possibly reflecting regional differences in pet-keeping habits (eg, pets might be kept outdoors in less urbanized areas).^23^^,^^24^ Practice parameters and guidelines encourage pet removal from homes of patients with atopic asthma because numerous studies have shown that exposures to pet allergens exacerbate asthma in pet-sensitized individuals.^6^^,^^25^ Compelling evidence for this recommendation was recently presented in a large, national study that estimated that among pet-sensitive patients with asthma, more than 1,700,000 excess asthma attacks and nearly 700,000 excess asthma emergency department/urgent care visits are associated annually with elevated pet allergen levels in the bedroom.^26^ Further research, however, is warranted, as noted in the recent NAEPP guideline update; only a few small-scale studies have examined the effects of pet removal, and results have been inconclusive.^18^^,^^27^

Although clinicians’ assessment of asthma triggers is an important part of self-management education and an integral part of asthma care,^28^, ^29^, ^30^ environmental management of asthma in practice is challenging. Avoidance and control measures often require labor-intensive approaches and/or lifestyle changes, which may affect the acceptance and use of these measures.^31^ Because previous studies have reported mixed findings on the extent to which clinicians’ assessment and advice on environmental triggers affect asthma outcomes,^32^ it is not surprising that recommendations to patients vary, and environmental control strategies tend to be underutilized.^33^ Yet patient education has been shown to be essential for successful asthma management.^6^ Although the importance of AAPs for patient education is highlighted in the EPR-3 and in the implementation guidelines,^6^^,^^34^ studies indicate that a large proportion of asthma patients may never receive a written plan on how to effectively manage their asthma and recognize and respond to exacerbations.^35^, ^36^, ^37^, ^38^, ^39^ The NAS was no exception; approximately half of the primary care and advanced practice providers reported using AAPs only sometimes or never. Frequent AAP use, however, was consistently associated with providing advice on avoidance of environmental asthma triggers. Our findings underscore the benefits of using AAPs, although some studies have challenged the usefulness of these plans.^40^^,^^41^ Personalized AAPs not only help patients manage their asthma but may also remind clinicians to discuss relevant environmental control measures during patient visits. Some studies suggest that increased use of electronic health records and various electronic decision support tools, including electronic AAPs, may positively affect guideline implementation and improve asthma self-management.^35^^,^^42^, ^43^, ^44^ Patient counseling is important for asthma self-management and can affect patient adherence to recommended treatments and practices, including implementation of environmental control measures.^31^^,^^45^^,^^46^

In contrast to the findings on the use of AAPs and asthma control recommendations, guideline adherence and self-efficacy in managing asthma were less consistently associated with implementation of the EPR-3 recommendations regarding environmental control. However, strong agreement with key guideline recommendations increased the odds of recommending avoidance of air pollution among primary care providers, while overall agreement with EPR-3 recommendations remained low among all clinician groups. Primary care clinicians with high self-efficacy were also more likely to assess environmental triggers and provide advice on cooking appliances. Our results suggest that group-specific interventions and strategies may offer opportunities to improve guideline adherence and compliance with environmental recommendations, but further research is warranted to expand understanding of differing practice patterns between clinician groups.

Our study has limitations. Low response rates in physician surveys,^47^^,^^48^ including this one, are common and may introduce nonresponse bias. However, a NCHS report that evaluated whether lower response rates and changes in the 2012 NAMCS design and implementation affected physician-level estimates showed no or minimal bias.^49^ Because patient visit data could not be linked to the NAS data,^9^ only limited information on patient characteristics was available. On the other hand, surveys that require access to records present higher burden to clinicians and tend to further reduce response rates.^48^ Lastly, the study findings are based on data collected in 2012 and reflect the implementation of the EPR-3 guidelines, which were published in 2007. A recently disseminated update to these guidelines^18^ offers more focused recommendations regarding indoor allergen mitigation in management of asthma and may help increase clinician implementation.

Despite these limitations, this study has several major strengths. The study population is a nationally representative sample of office- and CHC-based clinicians, making the results generalizable to asthma care providers in the United States. The NAS provides detailed data on assessment of adherence to environmental control recommendations and trigger avoidance, and also on clinician and practice characteristics, self-efficacy, and self-reported adherence to other key components in the EPR-3 guidelines. The findings offer insight into clinicians’ decisions and strategies regarding environmental control measures and identify areas where guideline uptake was suboptimal. Several studies have shown that guideline adherence as well as clinician perceptions and practices influence the effectiveness of asthma management.^50^^,^^51^

In summary, nationally representative clinician-reported data demonstrated that implementation of guideline recommendations on environmental management of asthma varied by clinician group and that group-specific studies might inform targeted guideline implementation interventions in the future. However, high implementation of other key components of the guidelines was associated with higher clinician compliance with environmental recommendations outlined in the EPR-3 guidelines, which suggests that increasing implementation of core guideline elements, such as assessing asthma control, may positively affect implementation of other guideline recommendations.

**Key messages**

• Implementation of guideline recommendations on environmental management of asthma varies by clinician group.
• Increasing implementation of other core elements of the asthma guidelines may positively affect clinician compliance with environmental recommendations.

## Disclosure Statement

Supported in part by the Intramural Research Program of the 10.13039/100000002National Institutes of Health, the 10.13039/100000066National Institute of Environmental Health Sciences (Z01-ES-025041), and through a contract to Social & Scientific Systems funded by the National Institute of Environmental Health Sciences (HHSN273201600002I). The findings and conclusions in this report are those of the authors and do not necessarily represent the official position of the Centers for Disease Control and Prevention or the US Environmental Protection Agency.

Disclosure of potential conflict of interest: The authors declare that they have no relevant conflicts of interest.

## Supplementary data

Supplementary Fig E1

Supplementary Tables E1-E4

Supplementary Table E5

Supplementary Table E6

Supplementary Table E7

Supplementary Table E8

Supplementary Table E9

Supplementary Table E10

Supplementary Table E11
