# Supplementary material for: Environmental management of asthma in clinical practice: Results from the 2012 National Ambulatory Medical Care Survey
Source: J Allergy Clin Immunol Glob. 2023 Nov 22;3(1):100192. doi: 10.1016/j.jacig.2023.100192 (PMC10770720; doi:10.1016/j.jacig.2023.100192)
Supplement: Supplementary Fig E1 [file mmc1.docx]

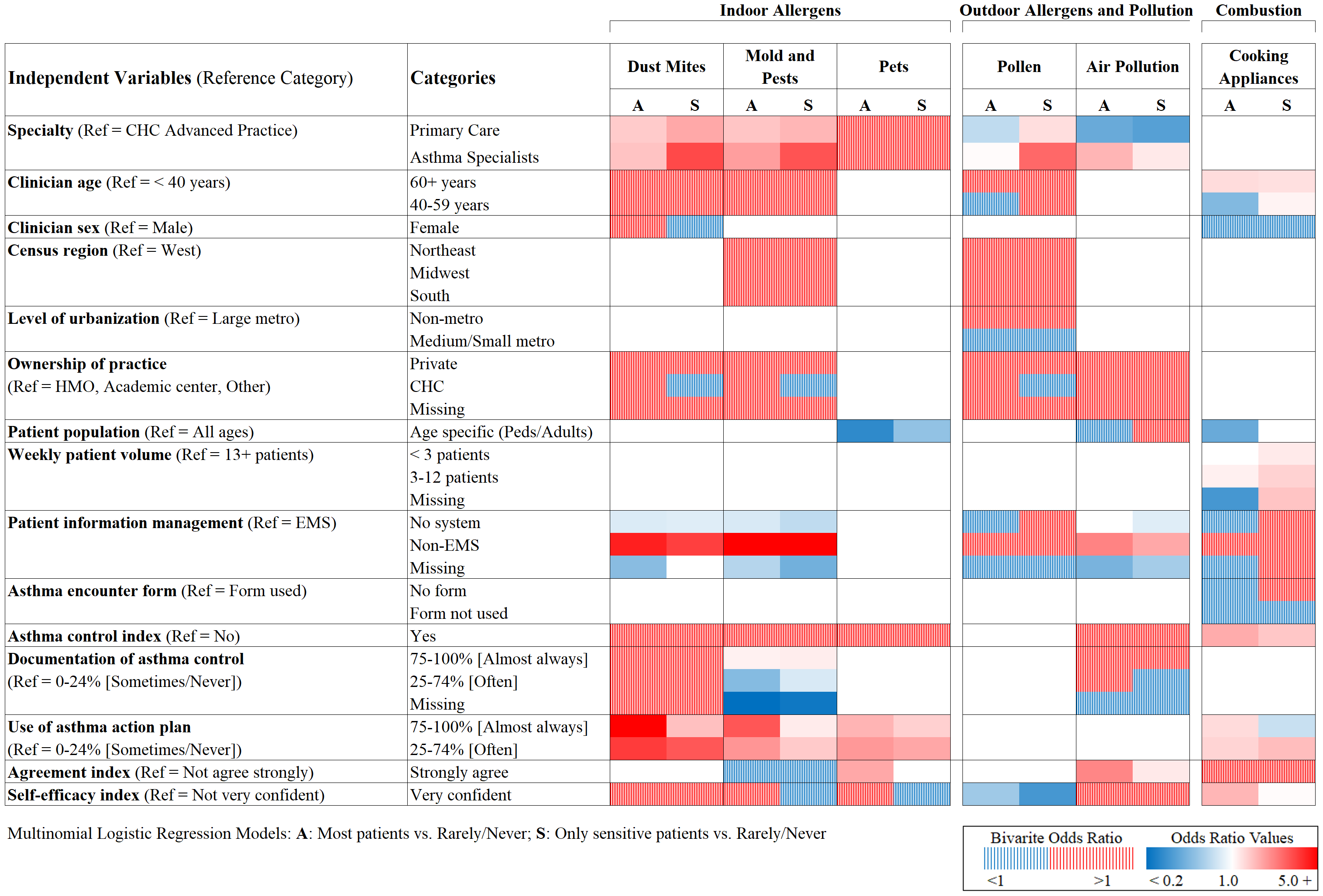


**Figure E1. Factors associated with guideline implementation: Environmental control recommendations among all clinicians.** The solid red/blue color indicates the direction of the associations (red positive; blue negative; P<0.05) when each of the clinician/practice characteristic categories were compared with the reference category adjusting for the other independent variables in the final models. The dashed areas indicate clinician/practice characteristics included in initial full models based on bivariate analysis results.

CHC Community health center; HMO Health maintenance organization
