# Supplementary material for: Environmental management of asthma in clinical practice: Results from the 2012 National Ambulatory Medical Care Survey
Source: J Allergy Clin Immunol Glob. 2023 Nov 22;3(1):100192. doi: 10.1016/j.jacig.2023.100192 (PMC10770720; doi:10.1016/j.jacig.2023.100192)
Supplement: Supplementary Tables E1-E4 [file mmc2.docx]

**Table E1. Categorization of questionnaire data by 4 key components of the EPR-3 guidelines^*^**

| **1. Assessment and monitoring of asthma severity and control^*^** | **Question** |
| --- | --- |
|  |  |
| **Assessment of impairment frequency**  For what percentage of asthma visits do you document overall asthma control?  For what percentage of asthma visits do you ask about patient’s ability to engage daily activities?  For what percentage of asthma visits do you ask about frequency of daytime symptoms?  For what percentage of asthma visits do you ask about frequency of nighttime awakening? | 7  8a  8b  8c |
| For what percentage of asthma visits do you ask about patient’s perception of symptom control?  For what percentage of asthma visits do you use control assessment tool (e.g., Asthma Control Test, Asthma Control Questionnaire, Asthma Therapy Assessment Questionnaire, etc.)?  For what percentage of asthma visits do you ask about frequency of rescue inhaler use (e.g., Albuterol)? | 8d  8e  8f |
|  |  |
| **Assessment of risk frequency** |  |
| For what percentage of asthma visits do you ask about frequency of emergency department visits or urgent care visits for asthma?  For what percentage of asthma visits do you ask about frequency of exacerbations requiring oral steroids? | 8h  8g |
|  |  |
| **Objective assessment and monitoring**  For what percentage of asthma visits do you ask about patient’s peak flow results from home?  For what percentage of asthma visits do you perform spirometry (among those who can perform spirometry)? | 8i  8j |
|  |  |
| **Ongoing monitoring frequency**  For what percentage of asthma visits do you assess daily use of controller medication (e.g., ICS) for patients with severe asthma?  For what percentage of asthma visits do you perform repeated assessment of inhaler technique? | 9g  9h |
|  |  |
| **2. Patient education^*^** |  |
| **Asthma action plans**  For what percentage of asthma visits do you provide a new or review an existing written asthma action plan outlining medications, triggers, and when to seek emergency care?  **Asthma therapies**  How often do you encounter patient misunderstandings about medication risks or side effects or belief in myths (e.g., muscle development, addiction)?  How often do you encounter patient concerns about short-term side effects of inhaled corticosteroids (e.g., thrush)?  How often do you encounter patient concerns about long-term side effects of inhaled corticosteroids (e.g., delayed growth in children)?  How often do you encounter confusion between symptom relief medications and daily controller medications? | 9a  13a  13b  13c  13d |
| **3. Assessment and control of environmental factors** |  |
| For what percentage of asthma visits do you assess triggers at home (e.g., pets, mold, tobacco smoke)?**^*^**  For what percentage of asthma visits do you assess triggers at school or workplace (e.g., mold, dust, exhaust, fumes, chemicals)?**^*^**  For what percentage of asthma visits do you test allergic sensitivity via skin or allergen-specific IgE (e.g., RAST) testing?**^*^**  For the following 7 questions, under what circumstances do you make the following recommendations about environmental exposures: 1) Most asthma patients, 2) Only patients with sensitivity to this trigger, or 3) Rarely or never recommend:  Using dust mite control measures (e.g., mattress covers)?  Controlling household mold and pests (e.g., cockroaches)?  Removing pets from home?  Avoiding pollen (e.g., limit outdoor time, close windows)?  Avoiding air pollution (e.g., ozone warnings)?  Making changes to cooking appliances (e.g., exhaust vents)?  Avoiding second-hand smoke? | 9b  9c, 9e  9f  10a  10b  10c  10d  10e  10f  10e |
| **4. Pharmacologic treatment** |  |
| Do you use the following medications for: 1) Symptom relief/acute exacerbation, 2) Daily long-term control, 3) Add on daily control therapy, 4) Difficult to control asthma, 5) Never use?  Short acting beta agonists  Inhaled corticosteroids  Long acting beta agonists (LABA)  Combination medication that includes both LABA and ICS  Leukotrine modifiers  Anticholinergics  Methylxanthines  Omalizumab  Short course of oral/injectable corticosteroids  Long course of oral corticosteroids (> 10 days) | 11a  11b  11c  11d  11e  11f  11g  11h  11i  11j |

* Adherence categories: Almost always (≥75% of the time), Often (25-<75% of the time), Sometimes (1%-24% of the time), Never (0% of the time)

2012 Asthma Supplement Questionnaire is available at: <https://www.cdc.gov/nchs/data/ahcd/2012_NAMCS_Asthma_Supplement.pdf>

Source: National Center for Health Statistics, 2012 National Asthma Survey of Physicians: National Ambulatory Medical Care Survey

**Table E2. Agreement and self-efficacy with the EPR-3 guidelines**

| **A. Assessment of agreement^*^** | **Question** |
| --- | --- |
| Spirometry is an essential component of a clinical evaluation for asthma diagnosis in patients able to perform it (please do not include peak flow monitoring as spirometry)  Inhaled corticosteroids are the most effective medications to control persistent asthma  Asthma action plans are an effective tool to guide patient self-management efforts  Patients with persistent asthma should have follow-up visits at least every 6 months to assess control  Assessing asthma severity is necessary to determine initial therapy | 5a    5b  5c  5d  5e |
| **B. Assessment of self-efficacy^†^** |  |
| Using spirometry data as a component of a clinical evaluation for an asthma diagnosis in patients  Assessing underlying asthma severity using standard criteria  Prescribing the appropriate dose of inhaled corticosteroids  Evaluating the need to step up controller therapy  Evaluating the need to step down controller therapy | 6a  6b  6c  6d  6e |

* Agreement categories: Strongly agree, agree, neutral, disagree, strongly disagree

† Self-efficacy categories: Very confident, somewhat confident, not at all confident, do not perform

2012 Asthma Supplement Questionnaire is available at: <https://www.cdc.gov/nchs/data/ahcd/2012_NAMCS_Asthma_Supplement.pdf>

Source: National Center for Health Statistics, 2012 National Asthma Survey of Physicians: National Ambulatory Medical Care Survey

| **Table E3. Environmental trigger assessment during asthma visits, 2012 NAS (weighted percentages; n =1,645)** | | | | | |
| --- | --- | --- | --- | --- | --- |
| **Clinician Assessment and Reported Frequency** | **All Clinicians** | **Primary Care** | **Asthma Specialists** | **CHC Advanced Practice** | ***P*-Value^*^** |
|  | **% (SE)** | **% (SE)** | **% (SE)** | **% (SE)** |  |
|  |  |  |  |  |  |
| Triggers at home |  |  |  |  | <0.001 |
| Almost always (75-100%) | 40.6 (2.2) | 39.6 (2.5) | 58.7 (4.2) | 38.5 (4.9) |  |
| Often (25-74%) | 41.0 (2.2) | 41.1 (2.5) | 35.2 (4.2) | 44.3 (4.5) |  |
| Sometimes/Never (0-24%) | 17.0 (1.7) | 17.7 (1.9) | 6.1 (2.2)^†^ | 16.7 (3.7) |  |
| Missing | 1.5 (0.5)^†^ | 1.6 (0.5)^†^ | 0.0 (0.0) | 0.5 (0.4)^†^ |  |
|  |  |  |  |  |  |
| Triggers at school/work |  |  |  |  | <0.001 |
| Almost always (75-100%) | 38.6 (2.2) | 37.6 (2.5) | 69.1 (4.0) | 28.4 (4.2) |  |
| Often (25-74%) | 36.6 (2.0) | 36.6 (2.3) | 27.5 (4.0) | 42.7 (4.6) |  |
| Sometimes/Never (0-24%) | 23.1 (1.9) | 24.0 (2.1) | 2.6 (1.2)^†^ | 28.5 (4.8) |  |
| Missing | 1.6 (0.5)^†^ | 1.8 (0.5) | 0.8 (0.8)^†^ | 0.5 (0.4)^†^ |  |
|  |  |  |  |  |  |
| Triggers at home and school/work^‡^ |  |  |  |  | <0.001 |
| Almost always (75-100%) | 30.3 (2.1) | 29.4 (2.4) | 53.6 (4.2) | 23.7 (3.9) |  |
| Often/Sometimes/Never (0-74%) | 69.7 (2.1) | 70.6 (2.4) | 46.4 (4.2) | 76.3 (3.9) |  |
|  |  |  |  |  |  |
| Objective assessment (sIgE, SPT)^§^ |  |  |  |  | <0.001 |
| Almost always (75-100%) | 8.5 (1.3) | 7.3 (1.5) | 35.0 (3.6) | 3.0 (1.6)^†^ |  |
| Often (25-74%) | 17.8 (1.6) | 17.5 (1.9) | 32.3 (4.1) | 11.6 (3.3) |  |
| Sometimes/Never (0-24%) | 70.4 (2.0) | 71.6 (2.3) | 32.8 (4.0) | 83.5 (3.6) |  |
| Missing | 3.3 (0.9) | 3.6 (1.0) | 0.0 (0.0) | 2.0 (1.3)^†^ |  |
|  |  |  |  |  |  |
| Review of triggers in AAP^¶^ |  |  |  |  | <0.001 |
| Almost always (75-100%) | 16.9 (1.5) | 15.6 (1.7) | 30.6 (3.6) | 21.2 (4.2) |  |
| Often (25-74%) | 30.3 (2.0) | 30.3 (2.3) | 32.7 (4.1) | 28.9 (4.2) |  |
| Sometimes/Never (0-24%) | 51.2 (2.2) | 52.4 (2.5) | 36.7 (4.4) | 49.3 (4.9) |  |
| Missing | 1.6 (0.5)^†^ | 1.8 (0.5) | 0.0 (0.0) | 0.7 (0.4)^†^ |  |
|  |  |  |  |  |  |
| * Χ^2^ test for difference between the clinician groups | |  |  |  |  |
| † Standard error > 30% (unreliable estimate) | |  |  |  |  |
| ‡ The variable combines the assessment of asthma triggers at home, school, and work to reflect a thorough assessment of triggers  during asthma visits | | | | |  |
| § Serum specific IgE, skin prick test |  |  |  |  |  |
| ¶ Asthma action plan |  |  |  |  |  |
| Source: National Center for Health Statistics, 2012 National Asthma Survey of Physicians: National Ambulatory Medical Care Survey | | | | | |

| **Table E4. Recommended environmental control practices, 2012 NAS (weighted percentages; n =1,645)** | | | | | |
| --- | --- | --- | --- | --- | --- |
| **Recommended Practices and**  **Patient Target Group** | **All Clinicians** | **Primary Care** | **Asthma Specialists** | **CHC Advanced Practice** | ***P*-Value^*^** |
|  | **% (SE)** | **% (SE)** | **% (SE)** | **% (SE)** |  |
|  |  |  |  |  |  |
| Indoor environment |  |  |  |  |  |
| Dust mite control measures |  |  |  |  | 0.045 |
| Most asthma patients | 39.7 (2.2) | 39.8 (2.4) | 36.1 (3.8) | 40.7 (4.6) |  |
| Patients with sensitivities | 46.4 (2.2) | 46.7 (2.5) | 56.2 (4.0) | 36.6 (4.4) |  |
| Rarely/never recommend | 11.8 (1.4) | 11.4 (1.5) | 6.7 (2.3) | 20.1 (4.0) |  |
| Control of mold and pests |  |  |  |  | 0.038 |
| Most asthma patients | 43.2 (2.2) | 43.1 (2.5) | 45.9 (4.3) | 42.5 (4.8) |  |
| Patients with sensitivities | 41.9 (2.2) | 42.3 (2.5) | 46.1 (4.3) | 34.3 (4.1) |  |
| Rarely/never recommend | 12.6 (1.6) | 12.1 (1.8) | 5.9 (2.2) | 21.6 (4.0) |  |
| Missing | 2.3 (0.5) | 2.4 (0.6) | 2.1 (1.4)^†^ | 1.6 (0.8)^†^ |  |
| Pet removal |  |  |  |  | 0.111 |
| Most asthma patients | 27.8 (2.0) | 27.3 (2.2) | 29.0 (3.9) | 32.7 (4.7) |  |
| Patients with sensitivities | 58.2 (2.2) | 59.2 (2.4) | 58.2 (4.1) | 47.8 (4.6) |  |
| Rarely/never recommend | 11.3 (1.3) | 10.7 (1.5) | 11.8 (2.4) | 17.9 (3.9) |  |
| Missing | 2.6 (0.6) | 2.8 (0.7) | 1.1 (1.0)^†^ | 1.6 (0.8)^†^ |  |
|  |  |  |  |  |  |
| Outdoor environment |  |  |  |  |  |
| Avoidance of pollen |  |  |  |  | 0.064 |
| Most asthma patients | 40.6 (2.1) | 41.3 (2.5) | 36.7 (4.3) | 54.6 (4.4) |  |
| Patients with sensitivities | 46.9 (2.1) | 47.5 (2.5) | 54.5 (4.3) | 35.0 (4.1) |  |
| Rarely/never recommend | 8.8 (1.4) | 8.4 (1.7) | 7.2 (1.9) | 8.8 (3.1)^†^ |  |
| Missing | 3.7 (0.8) | 2.8 (0.8) | 1.7 (1.2)^†^ | 1.6 (0.8)^†^ |  |
| Avoidance of air pollution |  |  |  |  | <0.001 |
| Most asthma patients | 44.9 (2.2) | 43.6 (2.5) | 62.4 (4.2) | 46.7 (4.9) |  |
| Patients with sensitivities | 32.7 (2.0) | 32.3 (2.3) | 29.6 (4.1) | 38.1 (4.9) |  |
| Rarely/never recommend | 20.3 (1.9) | 21.9 (2.2) | 6.7 (1.9) | 12.7 (2.6) |  |
| Missing | 2.2 (0.5) | 2.2 (0.6) | 1.3 (1.1)^†^ | 2.5 (0.9)^†^ |  |
|  |  |  |  |  |  |
| Combustion products |  |  |  |  |  |
| Change of cooking appliances |  |  |  |  | 0.628 |
| Most asthma patients | 16.1 (1.6) | 15.6 (1.8) | 21.5 (3.5) | 18.0 (3.9) |  |
| Patients with sensitivities | 30.2 (2.1) | 29.9 (2.4) | 33.2 (4.0) | 31.3 (4.3) |  |
| Rarely/never recommend | 51.6 (2.2) | 52.3 (2.5) | 44.2 (4.2) | 48.8 (4.8) |  |
| Missing | 2.1 (0.5) | 2.2 (0.6) | 1.1 (1.0)^†^ | 1.9 (0.8)^†^ |  |
| Avoidance of ETS |  |  |  |  | 0.676 |
| Most asthma patients | 81.8 (1.8) | 81.6 (2.0) | 84.5 (3.3) | 81.9 (3.2) |  |
| Patients with sensitivities | 12.0 (1.4) | 12.0 (1.6) | 12.6 (3.2) | 11.1 (2.6) |  |
| Rarely/never recommend | 4.1 (1.1) | 4.2 (1.2) | 1.8 (0.8)^†^ | 5.0 (1.8) |  |
| Missing | 2.2 (0.5) | 2.3 (0.6) | 1.1 (1.0)^†^ | 2.1 (0.9)^†^ |  |
|  |  |  |  |  |  |
| * Χ^2^ test for difference between the clinician groups | |  |  |  |  |
| † Standard error > 30% (unreliable estimate) | |  |  |  |  |
| Source: National Center for Health Statistics, 2012 National Asthma Survey of Physicians: National Ambulatory Medical Care Survey | | | | | |
